# Metabolomics to Understand Alterations Induced by Physical Activity during Pregnancy

**DOI:** 10.3390/metabo13121178

**Published:** 2023-11-29

**Authors:** Ana Carolina Rosa da Silva, Anahita Yadegari, Velislava Tzaneva, Tarushika Vasanthan, Katarina Laketic, Jane Shearer, Shannon A. Bainbridge, Cory Harris, Kristi B. Adamo

**Affiliations:** 1 School of Human Kinetics, Faculty of Health Science, University of Ottawa, Ottawa, ON K1N 6N5, Canada; arosada2@uottawa.ca (A.C.R.d.S.);; 2 Department of Chemistry and Biology, Toronto Metropolitan University, Toronto, ON M5G 2A7, Canada; 3 Alberta Children’s Hospital Research Institute, Cumming School of Medicine, University of Calgary, Calgary, AB T2N 4N1, Canada; 4 Department of Biochemistry and Molecular Biology, Faculty of Kinesiology, Cumming School of Medicine and Alberta Children’s Hospital Research Institute, University of Calgary, Calgary, AB T2N 1N4, Canada; 5 Interdisciplinary School of Health Sciences, Faculty of Health Sciences, Ottawa, ON K1N 6N5, Canada; 6 Department of Cellular and Molecular Medicine, Faculty of Medicine, University of Ottawa, Ottawa, ON K1H 8M5, Canada; 7 Department of Biology, University of Ottawa, Ottawa, ON K1N 6N5, Canada; charris@uottawa.ca

**Keywords:** pregnancy, physical activity, metabolomics, mass spectrometry, nuclear magnetic resonance

## Abstract

Physical activity (PA) and exercise have been associated with a reduced risk of cancer, obesity, and diabetes. In the context of pregnancy, maintaining an active lifestyle has been shown to decrease gestational weight gain (GWG) and lower the risk of gestational diabetes mellitus (GDM), hypertension, and macrosomia in offspring. The main pathways activated by PA include BCAAs, lipids, and bile acid metabolism, thereby improving insulin resistance in pregnant individuals. Despite these known benefits, the underlying metabolites and biological mechanisms affected by PA remain poorly understood, highlighting the need for further investigation. Metabolomics, a comprehensive study of metabolite classes, offers valuable insights into the widespread metabolic changes induced by PA. This narrative review focuses on PA metabolomics research using different analytical platforms to analyze pregnant individuals. Existing studies support the hypothesis that exercise behaviour can influence the metabolism of different populations, including pregnant individuals and their offspring. While PA has shown considerable promise in maintaining metabolic health in non-pregnant populations, our comprehension of metabolic changes in the context of a healthy pregnancy remains limited. As a result, further investigation is necessary to clarify the metabolic impact of PA within this unique group, often excluded from physiological research.

## 1. Introduction

A sedentary lifestyle is a global health concern and a significant risk factor for mortality worldwide. In contrast, the recognition of the benefits of regular exercise dates back to Hippocrates and is recommended to reduce the risk of health conditions like diabetes, cancer, and obesity [1,2,3]. Metabolomics, a cutting-edge discipline in the field of systems biology, focuses on comprehensively analyzing the small-molecule metabolites present within biological systems and has gained popularity in characterizing physiological responses to exposures like physical activity (PA). Techniques such as liquid chromatography–mass spectrometry (LC-MS), gas chromatography–mass spectrometry (GC-MS), and proton nuclear magnetic resonance (1H NMR) are commonly employed for compound annotation. By identifying and quantifying an array of metabolites, metabolomics enables a comprehensive exploration of the metabolic signatures associated with various physiological states, thus offering insights into the mechanisms through which exercise impacts cardiometabolic health, including glycemic control, blood pressure regulation, and lipid profiles. For example, PA is positively associated with the amino acids glycine and citrulline [1], while exercise-induced changes in phospholipids, oxylipins, carnitines, and amino acids have been observed in the plasma of non-pregnant women within reproductive age. Additionally, decreases in branched-chain amino acids, glycine, citrulline, and 1-methyladenosine, have been linked to type 2 diabetes (T2D) [1,4].

Habitual PA is a behaviour known to support a healthy pregnancy. Evidence-based guidelines recommend engaging in at least 150 min of moderate-intensity PA per week during pregnancy [5] due to its potential to reduce the risk of gestational diabetes mellitus (GDM) (↓ 38%), gestational hypertension (↓ 39%), and macrosomia (↓ 39%) without the likelihood of preterm birth, low birth weight, or growth-restricted babies [6,7]. Incorporating a variety of aerobic and resistance training activities, such as yoga and/or gentle stretching, has been shown to be beneficial [8]. Higher sedentary behaviour during pregnancy is positively associated with higher levels of C-reactive protein, LDL cholesterol, and macrosomia [9]. Research has emphasized the positive health outcomes for offspring when their birthing parents embrace healthly habits during pregnancy [5,8]. For example, regular PA has been shown to decrease cord blood cholesterol levels in babies born to active and healthy gestational parents; thus, exercise is thought to influence the intrauterine environment [10]. Likewise, in offspring from parents who exercised during pregnancy, metabolites such as tyrosine and phenylalanine were increased while lactate decreased, suggesting an impact of PA on fetal amino acid metabolism [11]. Moreover, branched-chain amino acids (BCAAs) decreased in the urine of pregnant women after PA, while serum levels of polyunsaturated fatty acids increased in gestational parents with obesity [12,13]. These metabolites are intermediates or cellular metabolism products, and their identification might improve our understanding of the effects of PA on human health and disease. Therefore, the metabolomics approach has risen in popularity to explore associations between various exposures and low-molecular-weight metabolites (e.g., carbohydrates, amino acids, and organic acids) or lipidomics, focusing on the analysis of lipids in a biological sample [2]. Both metabolomics and lipidomics have been instrumental in studying the effects of PA due to their high-throughput capabilities, sensitivity, specificity, and coverage of a broad range of metabolite classes. Several studies have shown that PA, acute bouts of exercise, and sedentary time can significantly modify the metabolic profile of the host. These changes are most observed in lipids, amino acid (AA), the tricarboxylic acid (TCA) cycle, insulin sensitivity, and glycolysis-related metabolic pathways [1,14,15].

This narrative review discusses the impact of PA on metabolite profiles with a specific focus on pregnant individuals. A comprehensive search of Google Scholar, PubMed, and Scopus up to April 2023 was conducted. The inclusion criteria were as follows: animal studies, observational or interventional articles; non-athletic pregnant or non-pregnant populations; populations of a reproductive age; overweight individuals; people with obesity with or without metabolic syndromes; written in English, French, Persian, and Portuguese. A complementary search was also performed by scanning the reference lists of the eligible articles to identify additional studies. Overall, a total of 28 articles were identified for non-pregnant (*n* = 20) and pregnant (*n* = 8) populations from 2014 to 2023 and included in the review.

## 2. Effects of PA in Non-Pregnant Populations Using Metabolomics

The literature has shown that distinct levels of PA induce changes in the metabolome, which MS or NMR can assess. Alterations in the metabolites involved in the AA, phospholipid (PL), and carnitine metabolisms, as well as the TCA cycle, are some examples of the most characterized thus far.

### 2.1. Changes in Amino Acids

AAs hold a pivotal role in human biology, and their functions extend beyond mere involvement in protein synthesis. These metabolites play vital roles in different pathways in living organisms, such as cell division, redox status, glycogenolysis, and lipolysis. Their concentration and regulation significantly impact metabolic health, with deviations from normal levels indicating potential pathophysiological conditions [16]. AAs are divided into several subgroups based on their structural and functional characteristics. The main categories linked to PA in the literature are branch-chain amino acids (BCAAs) (leucine, isoleucine, and valine), aromatic amino acids (AAAs) (phenylalanine, tyrosine, and tryptophan), sulfur-containing AAs (methionine, cysteine, and cystine), AAs involved in the uric acid cycle (arginine, ornithine, and citrulline), gluconeogenic AAs (alanine and glutamine), and AAs involved in the glycine–serine–threonine metabolic axis. Each of these major categories plays an important role in metabolism, as summarized in Table 1. This section explores connections between these diverse AA subgroups and PA, with an emphasis on their roles in cardiometabolic well-being.

One of the major trends reflected in both observational and interventional studies is the inverse association between PA and/or total branched-chain amino acid (BCAA) levels [4]. A lower mitochondrial capacity, previously linked to obesity, T2D, and aging, is known to impair the metabolism of BCAAs, resulting in their accumulation [30]. Elevated plasma and tissue BCAAs, especially leucine, can lead to the overstimulation of the mTOR/p70S6K pathway. This pathway plays key roles in protein synthesis, cell growth, proliferation, migration, and normal development. However, its dysregulation can interfere with insulin functions and cause hepatic insulin resistance (IR). Furthermore, BCAA transamination products are catabolized into TCA cycle intermediates, acetyl-CoA and succinyl-CoA, leading to anaplerotic stress and destabilizing metabolic function [31]. Consistent with this overstimulation paradigm, IR, diabetes, and obesity have all been associated with higher BCAA levels. Xiao and colleagues reported an inverse association between PA energy expenditure (PAEE)—calculated from accelerometry data—and plasma valine and isoleucine [14]. Comparable to these findings, a study by Vanweert et al. showed that when stratified by PA levels obtained from self-reported questionaries, plasma isoleucine and leucine were significantly higher in less-active individuals compared to more-active individuals [4]. Similarly, in a cross-sectional study in 2022, Hamaya et al. reported that total plasma BCAAs and leucine levels were inversely associated with total leisure-time physical activity (LTPA) when comparing the lowest LTPA quartile to the highest, even after adjusting for BMI. The LTPA was assessed through self-reported questionnaires and calculated as MET (metabolic equivalent of task) hours per week [32]. The existing literature highlights that PA can boost mitochondrial capacity [33]. One of the main proposed contributors to BCAA accumulation is suboptimal mitochondrial capacity; the inverse PA and BCAA association may be due to an increased mitochondrial capacity and, consequently, enhance BCAA catabolism.

Additionally, PA is negatively correlated with circulating levels of aromatic amino acids (AAAs)—phenylalanine, tyrosine, and tryptophan—and sulfur-containing amino acids—methionine, cysteine, and cystine [34,35]. AAAs are important proteogenic AAs and are key precursors for several biologically and neurologically active compounds such as dopamine, serotonin, norepinephrine, and epinephrine [36]. However, elevated levels have been linked to insulin resistance, lower insulin secretion, and a higher risk of developing T2D [37]. Furthermore, the tyrosine isomers meta-tyrosine and ortho-tyrosine have been characterized as oxidative biomarkers, suggesting that the accumulation of AAAs is also linked to oxidative damage [38]. Evidence suggests that insulin resistance (IR) is associated with altered sulfur amino acid tissue metabolism [37]. Elevated cysteine has been linked to obesity, metabolic syndrome, higher total fat mass, IR, inflammation, endothelial dysfunction, and the generation of reactive oxygen species (ROS) [37,39]. Greater levels of these AAs can interfere with the metabolism of BCAAs and AAAs via the inhibition of branched-chain α-keto acid dehydrogenase (BCKD) and tyrosine aminotransferase, respectively [40]. Kasperek and colleagues (2023) reported that a 6-week moderate-intensity aerobic exercise intervention upregulated the AAA metabolic pathways in sedentary adults, regardless of their obesity status [35]. Corroborating these findings, Grapov et al. (2019) [34] documented that among sedentary, insulin-resistant women with obesity partaking in a moderate-intensity exercise session, there was a reduction in plasma total and specific aromatic amino acids (AAAs), such as tryptophan and phenylalanine. The authors also reported a reduction in plasma methionine, cysteine, and cystine during the exercise sessions. However, post-recovery, none of the changes remained significant [34]. Given the relationship between AAAs and sulfur-containing AA accumulation and adverse markers of cardiometabolic health such as high-fat mass, IR, inflammation, oxidative stress, and endothelial dysfunction, their negative correlation with PA can offer several pathways through which PA might mitigate cardiometabolic disorders.

A noteworthy observation across the studies was that the circulating levels of AAs involved in the uric acid cycle—arginine, ornithine, and citrulline—were increased in response to PA [1,41,42]. Citrulline and arginine are precursors for nitric oxide synthesis, a molecule with important regulatory effects on endothelial function. In addition, arginine has also been linked to improved insulin sensitivity [43], and citrulline also displays anti-inflammatory properties [44]. Palmnäs et al. (2018), who studied the association between serum metabolites and PA (self-reported) in adults with normal weight and obesity, found higher levels of several metabolites, including arginine, in women with a normal weight who engaged in more PA [45]. Similarly, Babu et al. (2022) investigated the effects of a 12-week high-intensity interval training (HIIT) intervention on 13 women with metabolic-dysfunction-associated steatotic liver disease (MASLD), and post-intervention, several adipose tissue amino acids, including arginine, were increased [42]. In a recent cohort study led by Kojouri et al. (2023), vigorous PA assessed through self-reported questionnaires and measured as metabolic equivalent (MET) minutes per week was associated with a significant enrichment in the arginine and proline biosynthesis pathways [46]. Likewise, an observational study described a positive association between citrulline and self-reported PA [1]. Kuehnbaum et al. (2014) reported that a 6-week HIIT program significantly increased plasma ornithine levels in women with overweight or obesity [41]. The increase in arginine seen in the PA intervention groups may likely be a “standby” mechanism in preparation for quick nitric oxide (NO) release to enhance performance during exercise via its vasodilatory properties (e.g., improving blood flow to muscles) [47]. Given the functions that these AAs serve in enhancing IR and endothelial function and displaying anti-inflammatory attributes, their elevation as a response to exercise aligns with the concept that consistent physical activity contributes to cardiovascular well-being [43,44]. 

Alanine, glutamate, and glutamine are important gluconeogenic AAs linked to PA and metabolic health. Alanine is an important energy source for the central nervous system [24]. Glutamate is an important mediator of excitatory signals and nervous system plasticity [48]. However, alanine and glutamate accumulations are linked with conditions such as T2D, insulin resistance, and coronary heart disease [46]. In a nested case–control study by Pang and colleagues, higher plasma glutamine and lower plasma alanine were associated with higher self-reported PA and lower sedentary leisure time [49]. Greater PA was also linked with reduced plasma glutamate levels [49]. Similarly, an inverse relationship was observed between vigorous PA, calculated as weekly MET minutes, and plasma glutamate levels, even when accounting for BMI [46]. A study by Xiao and colleagues also highlighted a comparable unfavorable link between glutamate levels and physical activity energy expenditure (PAEE), obtained from accelerometry data [14]. The decrease in these AAs attributed to physical activity implies a possible route toward enhanced cardiometabolic health. Concurrently, elevated levels of circulating glutamine and an increased ratio of glutamine to glutamate prove advantageous, as they are correlated with a decreased risk of type 2 diabetes (T2D). Moreover, heightened glutamine levels contribute to the regulation of antioxidants, as glutamine acts as a precursor for synthesizing glutathione—a vital regulator of antioxidant properties within the body [50]. This positive correlation between physical activity and glutamine suggests an additional avenue for bolstering insulin sensitivity and reinforcing antioxidant capacity through exercise.

Circulating serine and glycine, as well as adipose tissue threonine, three interconnected AAs sharing common biochemical pathways, exhibit positive associations with PA. These AAs play essential roles in the development and functioning of the central nervous system [29]. Notably, Palmnäs et al. (2018) observed elevated serum serine in women physically active with a normal weight [45]. Moreover, research by Ding et al. highlighted a positive association between glycine and self-reported PA, even after adjusting for BMI [1]. Increased threonine levels in adipose tissue were also documented in response to a 12-week HIIT intervention by Babu and colleagues [42]. The positive associations between these AAs and PA underscore the potential cognitive and mental health benefits of heightened PA. The interplay of the serine–glycine–threonine axis with carbohydrate metabolism and antioxidant pathways suggests that PA contributes to enhancing carbohydrate utilization and bolstering antioxidant capacity [29]. Other findings regarding individual AAs and their relationship with PA include a positive association with circulating betaine and an inverse association with circulating aspartic acid [1,29,34,42,45]. The positive association between betaine and PA (self-reported), as identified by Palmnäs et al. (2018), aligns with the existing literature indicating that a higher circulating betaine level is linked with a more favorable body composition—characterized by reduced body fat and increased lean body mass [45]. Moreover, betaine supplementation has been shown to stimulate fatty acid β-oxidation and enhance insulin and insulin-like growth factor 1 receptor signaling, suggesting that the positive connection between betaine and PA can contribute to cardiometabolic health by improving lipid metabolism and insulin function [51]. Conversely, in the context of a HIIT session, there was a decrease in circulating aspartic acid in the intervention study by Grapov et al. (2019) [34]. The available data have linked higher levels of aspartic acid with IR, presenting an additional plausible pathway through which acute PA can enhance insulin sensitivity [52].

Table 2 summarizes relevant studies involving changes in amino acids in response to acute or habitual PA in non-pregnant populations. The discussed studies reveal a complex interplay between PA and AA metabolism. Collectively, the existing evidence reveals that an AA profile associated with a higher PA status is consistent with the profile characteristic of individuals with better cardiometabolic health, encompassing better glucose tolerance and endothelial function. These observations underscore the intricate routes through which PA can modulate AA metabolism, ultimately fostering a state of cardiometabolic well-being.

### 2.2. Changes in Lipids

Lipidomics is the study of lipids, a class of metabolites that have distinct cellular functions [54]. They are divided into subclasses according to their head group and the type of linkage between the head group and aliphatic chains. The main subclasses linked to PA in the literature were phospholipids (PLs), diacylglycerols (DGs), fatty acids (FAs), and acylcarnitines (ACs), and the reviewed literature is summarized in Table 3. 

Phosphatidylethanolamines (PEs) and phosphatidylcholines (PCs) are the most abundant PLs in human blood and tissues, and consequently, most studies focus on the associations between these classes and metabolic disorders [30]. Studies have demonstrated that decreased insulin sensitivity is associated with low concentrations of PC- and PE-containing polyunsaturated fatty acids (PUFA). Therefore, it might be suggested that fatty acid composition plays an important role in the action of insulin [30,55]. 

An investigation by Morris et al. examined the plasma lipid response of women (*n* = 20) to an acute exercise bout on a cycle ergometer. The regimen entailed a three-minute warmup, followed by four-minute workloads at intensity levels of 15, 35, 55, and 75% of VO_2_ max. Their outcomes revealed notable changes in PCs, PEs, and ceramides as the primary altered lipid classes after the exercise. Post-exercise, the levels of PE(34:3), PC(34:2), PC(36:4), and Cer(10:0) were increased [15]. In addition, Ding et al., 2019, described increased lipids after exercise training, especially lysophosphatidylcholines (LPCs), PCs, and PEs (e.g., LPC(18:1), PC(36:0), PC(34:3p), PE(38:3p)) [1]. Furthermore, Mendham et al., 2021, investigated the lipid composition of skeletal muscle in women with obesity after 12 weeks of supervised aerobic and resistance training. Overall, PA increased the content of PC(32:0), PC(34:2), and PI(38:4), as well as a particularly evident rise in PEs containing polyunsaturated fatty acids, such as PE(16:0/20:4) and PE(18:0/20:4). However, the levels of LPCs, like LPC(16:0) and LPC(18:2), exhibited a reduction after the training [56]. When exploring the plasma lipidome of women with obesity undergoing 8 weeks of supervised PA training, alternating between strength and aerobic exercises, a decline in the quantities of PC(40:6), PE(36:5), and SM(d18:1/20:0) was found [3]. It is well known that obesity increases the risk of insulin resistance; therefore, the increased concentration of UFAs within the phospholipid composition post-physical activity seen in this population may improve insulin resistance.

**Table 3:** Summary of lipidomics studies on the non-pregnant population.

Study Population and Sample Size	Biosample	Study Design	Metabolomic Profiling Platform	PA Intervention or Measurement	Main Results
214 people, including 20 women (18–65 years of age)	Plasma	Intervention	LC-MS	The test used a cycle ergometer for a three-minute warm-up at baseline, followed by four-minute steady-state workloads at 15, 35, 55, and 75% of VO_2_ max	PE(34:3), PC(34:2), PC(34:3), PC(36:3), and Cer(10:0) were some of the lipids higher after the exercise intervention [15].
14 obese sedentary individuals (5 women, 40 ± 2 years of age) and 14 persons with type 2 diabetes (5 women, 43 ± 2 years of age)	Skeletal muscle	Intervention	LC-MS	Cycle ergometer for 1.5 h at 50% of VO_2_ max	The total PE amount was higher in people with insulin resistance after a 90-min bout of PA [57].
12–15 women with obesity (30–50 years of age)	Plasma	Intervention	LC-MS	30 min of aerobic exercise for 4 days/wk at an intensity of 60–70% of maximal HR	Palmitoyl and oleoyl carnitines were slightly reduced after exercise [58].
5197 people, including 2120 healthy women (25–42 years of age)	Plasma	Cross-sectional	LC-MS	Self-reported questionnaires every 2–4 years	PA was positive associated with LPC(18:1), PC(36:0), PC(34:3p), PE(38:3p), CE(18:1), CE(16:0), and SM(22:1) and negative associated with DG(34:2) and TG(50:2) [1].
12–15 women with obesity (30–50 years of age)	Plasma	Intervention	LC-MS	Data from calorie-restricted diet + 14 wks of 4 day/wk aerobic exercise at 60–75% of maximal HR combined with data from an acute bout of exercise	Epoxides (19(20)-EpDPE, 13-HOTE, 11-HETE), prostaglandins (PGE1, PGE2, PGD2), and endocannabinoids (2-AG, AEA) were significantly reduced by physical training [34].
14 women with obesity (no exercise, 24 ± 4 years of age) and 19 obese women (exercise, 23 ± 3 years of age)	Skeletal muscle	Intervention	GC-MS AND LC-MS	12 weeks of combined aerobic (75–80% of peak HR) and resistance (60–70% of maximum HR) exercise training	Cardiolipins, PLs, and acylcarnitines increased, while LPC content decreased after PA [56].
20 women with obesity (35 ± 6 years of age)	Plasma	Intervention	LC-MS	8 weeks of aerobic and strength exercises, alternately, for 55 min at 75–90% of maximum HR, 3 times a week	Downregulated lipid species were PC(36:0), PC(40:6), PC(36:5p), PC(42:1p), PE(40:4p), PE(36:5), PE(38:3p), TG(54:8), TG(56:9), TG(60:11), SM(d18:1/20:0), and stearic acid, while upregulated lipid species were LPC(16:0p), LPC(18:0p), LPC(20:2), PC(20:2), TG(48:0), TG(50:0), and SM(d18:1/26:1) [3].
Adults who are sedentary; 14 lean and 10 obese	Plasma Stool	Intervention	LC-MS	6 wks of supervised, aerobic exercise 3×/wk (60–75% of heart rate reserve 30–60 min	Palmitic acid was significantly increased post-intervention [35].

**Figure 1:**
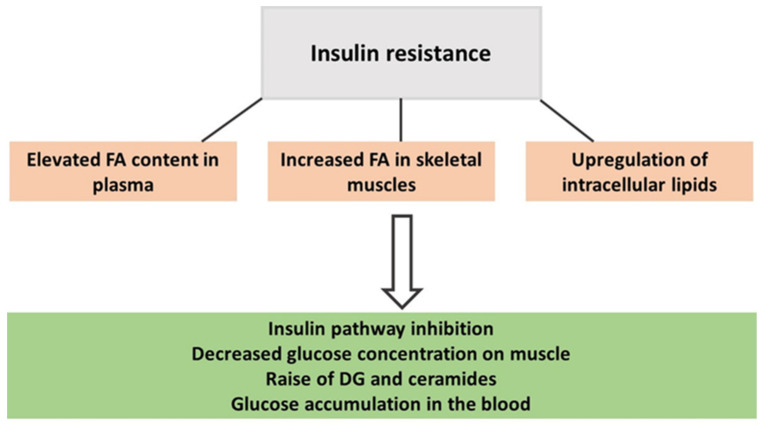
The onset of insulin resistance in people with obesity.

From mouse models, it has been described that the enzyme ethanolamine-phosphate cytidyltransferase is involved in PE production and can contribute to the accumulation of DGs and triacylglycerols in mouse tissue and impair insulin sensitivity [55]. IR reduces the muscle response to circulating insulin and is a state characterized by an elevated presence of toxic lipid intermediates such as DGs and ceramides (Figure 1) [59,60,61]. PA plays a critical role in redirecting dietary fats towards mitochondrial oxidation rather than promoting their storage, thereby enhancing fatty acid (FA) oxidation capacity and the expression of lipolytic proteins. Consequently, the content of those harmful lipids is reduced [59,60,61]. A lipidomics study found that greater volumes of self-reported weekly PA decreased DGs in the blood of healthy women [1], as did 8 weeks of supervised PA training alternating between strength and aerobic exercises in women with obesity. As a result, DG(34:0) and DG(36:0) were reduced by at least 20% in their plasma [3].

ACs such as Car(16:0) and Car(18:1) were slightly reduced after 14 weeks of supervised training [58], whereas Car(20:2) and Car(14:0) increased after 12 weeks of supervised aerobic and resistance training [56]. Long ACs have been positively associated with insulin resistance [62]. Medium and long ACs are also linked to cardiovascular diseases, which may be caused by altered mitochondrial fatty acid oxidation. A failing heart is characterized by poor FA oxidation, which leads to the accumulation of intermediates such as ACs [62]. 

Free FAs, as the building blocks of lipid metabolism, have also been investigated [54]. Mendham et al. described that omega-3 FA arachidonic acid concentrations rose [56], whereas Kasperek et al., 2021 reported a significant reduction in the palmitic acid levels of individuals who were sedentary and of a varying weight status (obese and lean), following an aerobic exercise intervention lasting 6 weeks [35]. FA composition was correlated with the skeletal muscle response to insulin and obesity, and the level of unsaturation was linked to insulin resistance [63].

This section summarizes some of the research studying whether the lipid profile is influenced by PA in non-pregnant individuals. Overall, PLs increased after a bout of exercise or aerobic training, while DGs decreased, and the AC concentration was inconsistent in the investigated literature. These findings show variability across studies, possibly due to the differences not only in sample extraction methods but also in analytical platforms. Furthermore, the type of exercise, duration, and intensity might differentially impact the human lipidome. Nonetheless, characterizing the lipids using an omics strategy may significantly improve our understanding of metabolic variations caused by PA.

### 2.3. Other Metabolites

Xiao et al. demonstrated that a greater PA intensity is inversely associated with the levels of 2-hydroxybutyrate (2-HB) and 3-hydroxybutyrate (3-HB) [14]. 2-hydroxybutyrate (2-HB) is a product of alpha-ketobutyrate and a known biomarker for insulin resistance and type 2 diabetes [14]. Likewise, 3-HB and formate were also higher in females after walking 150 min per week for 12 months [64]. Formate is an organic acid and an intermediate of acetate metabolism. Similarly, an exercise intervention performed by Kasperek et al. in 2023 also reported a significant post-exercise reduction in serum 3-HB levels in response to aerobic exercise performed 3×/week at 60–75% of heart rate reserve for 30–60-min, post-intervention, irrespective of obesity status [35]. Hence, its inverse correlation with physical activity could suggest enhanced lipid metabolism in individuals with healthier lifestyle habits [14]. 

Self-reported vigorous PA levels were directly associated with fumarate, which is the final product of the TCA cycle. Hence, its inverse correlation with physical activity could suggest enhanced lipid metabolism in individuals with more beneficial lifestyle behaviours [46]. Plasma levels of lactate, glucose, and acetoacetate were inversely associated with PA [49]. Other carbohydrates such as maltose, fructose, and glycerol 3-galactoside plummeted in a study conducted by Grapov et al. (2019) in which sedentary, insulin-resistant women with obesity participated in moderate-intensity exercise sessions [34]. Additionally, a pre–post blood-drop analysis following 30 min of running illustrated increases in glucose levels, while the lactate, pyruvate, succinate, and 2-HB amounts decreased [65]. Organic acids such as lactate and TCA cycle products are increased during physical effort and reduced during recovery [66]. 

Thiols are involved in the maintenance of homeostasis as well as the antioxidant defense system. For example, cysteine residues are temporary redox sensors due to their capacity to act as reducing agents [67,68]. The release of ROS in the body is related to their physiological role as signaling molecules. However, their increased production and/or insufficient performance of antioxidant systems can lead to oxidative stress, which is associated with the damage or/and oxidative modification of vital molecules such as nucleic acid, proteins (enzymes), and lipids [68]. Kuehnbaum et al. studied the effects of a 6-week HIIT training program in women who were overweight/obese and sedentary. The exercise intervention was able to significantly reduce glutathione–cysteine disulfide, cysteinyl glycine–cysteine disulfide, and cystine. These molecules are circulating oxidized thiols. Elevated plasma-oxidized disulfides are associated with diabetes; therefore, reduced levels of plasma thiol redox status post-exercise indicate a greater detoxification capacity [41]. Changes also included a downregulation in circulating glutathione–L-cysteine mixed disulfide, which could indicate higher antioxidant capacity. The exercise training program was also able to attenuate plasma hypoxanthine concentrations, which could be an indicator of lower energetic stress [69]. Modifications to these may constitute a mechanism through which PA improves antioxidant capacity, thereby reducing cardiovascular risk.

A recent study was able to confirm that a PA intervention could induce changes in xenometabolites [35]. The metabolome profile of serum and stool samples after a 6-week aerobic exercise intervention in adults who were sedentary (lean and obese) was investigated. The authors reported a rise in two specific aromatic microbial-derived amino acid metabolites in the blood: indole-3-lactic acid and 4-hydroxyphenyllactic acid. Interestingly, this surge was observed regardless of whether the participants were lean or obese, indicating that exercise is a more powerful stimulus than weight status. Indole-3-lactic acid is known for its anti-inflammatory properties and its ability to scavenge free radicals. It is also involved in inhibiting the production of interleukin-6, which is a pro-inflammatory cytokine. On the other hand, 4-hydroxyphenyllactic acid has been shown to reduce the generation of ROSs in white blood cells, specifically neutrophils [35]. As such, the measured changes are in line with PA mitigating/attenuating risks associated with metabolic diseases, although future research is needed to better comprehend the potential of xenometabolites in PA and their physiological pathways relevant to human health. 

Briefly, physical activity leads to changes in organic acids. For example, there is an increase in 2-HB, accompanied by a decrease in lactate post-exercise. Furthermore, there is a reduction in thiols, which are linked to the antioxidant system as they act as reducing agents. These findings highlight the diverse array of metabolite classes through which metabolomics has been delineating alterations attributed to physical activity. 

## 3. Effects of PA in Pregnant Populations Using Metabolomics

**Figure 2:**
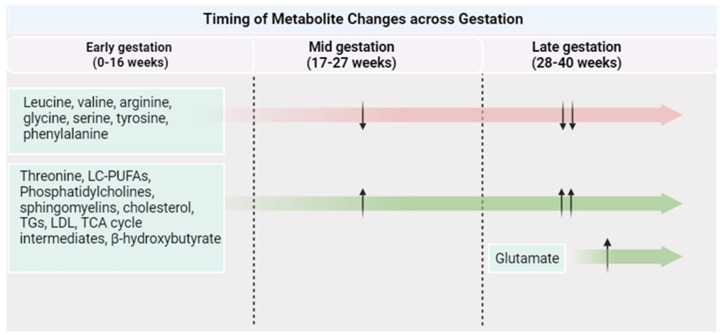
Metabolite changes across a healthy pregnancy. Created with Biorender.com (accessed on 1 October 2023).

Pregnancy itself is accompanied by vast physiological changes, such as reduced insulin sensitivity, enhanced ketogenesis during the fasting state, and higher lipid deposition [70]. These adaptations are vital to support fetal growth and development during gestation, as well as to provide the fetus with the necessary energy and nutrients following birth [71]. These physiological changes can give rise to changes in protein, fat, and carbohydrate metabolism and subsequently result in a distinct metabolome profile during gestation (Figure 2). Considering this, the metabolome response to PA can be different than that observed in the general population. 

**Figure 3:**
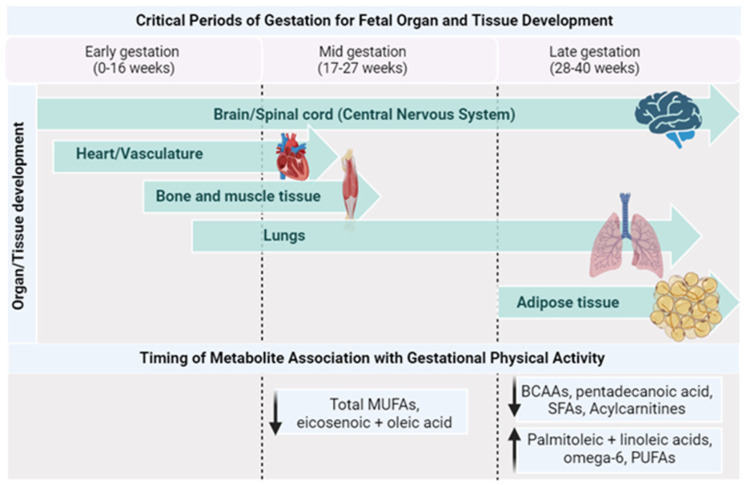
Timing of metabolite changes by PA with critical periods of gestation. Created with Biorender.com (accessed on 1 October 2023).

Table 4 outlines the studies examining relationships between exercise/PA and metabolomics during pregnancy. Most of the published literature in this field focuses on individuals with obesity or pregnancies with complications. The beneficial effects of PA are well established for gestational individuals and their offspring [6,8,72]; however, how PA affects their metabolite profile has not yet been elucidated. While data are limited, this section strives to provide a concise overview of the available literature that characterizes the metabolomic patterns of gestational parents and their offspring, with a particular focus on the impact of physical activity on these profiles (Figure 3).

As pregnancy progresses, there is an increase in the transfer of phospholipid FAs, particularly long-chain PUFAs, to the fetoplacental compartments [76]. The plasma FAs from the birthing parent, which result from both dietary intake and metabolic processes, serve various functions during pregnancy. For instance, they accumulate in the fetal nervous system and are necessary for its development [77], they can potentially influence parental inflammation [78], and they can have implications for the development of gestational diabetes [79,80]. PUFAs exert regulatory effects on various processes during early placental development, such as angiogenesis [81]. These PUFAs are supplied by the birthing parent to the fetus, with a significant impact on fetal brain development. This assimilation increases particularly during the third trimester [82]. Found predominantly in membrane phospholipids, PUFAs serve as vital structural components of the brain and retina, influencing fetal neurodevelopment and growth. They also play a crucial role in modulating neuronal signaling and the physical and functional properties of the membrane, including receptor function and membrane fluidity [83]. Insufficient PUFAs during fetal brain growth and development have the potential to affect brain maturation and plasticity and compromise its function in adult life [83]. Additionally, gestational parents’ PUFAs positively affect fetal growth, length, and birth weight [84]. A recent metabolomics study observed that abnormal fetal growth in late pregnancy was associated with lower levels of PUFAs [85]. In fact, PA lifestyle interventions in pregnant individuals with obesity were shown to improve their fatty acid profiles, highlighted by a higher concentration of beneficial FAs such as linoleic, omega 6, and other PUFAs, as well as lower concentrations of saturated fatty acids [13]. Higher levels of saturated fatty acids, specifically even-chain unsaturated fatty acids, are linked to higher lipotoxicity [86], impaired insulin sensitivity, glucose intolerance by triggering the proteasomal degradation of key insulin-signaling molecules [87], and stimulating proinflammatory signaling through the activation of Toll-like receptor 4 [88], while odd-chain saturated fatty acids such as pentadecanoic and heptadecanoic acid have shown protective effects against metabolic disorders [89,90]. In addition, monounsaturated FAs (MUFAs) were shown to be significantly higher in physically active pregnant individuals during mid-pregnancy compared to their inactive counterparts [91]. Recent work by Rafiq et al. explored the non-dietary factors that can lead to variations in circulating food-related metabolites during pregnancy, including PA. Their data from 600 pregnant individuals in mid-to-late gestation (24–36 weeks) revealed that higher PA levels were linked with lower serum pentadecanoic acid, an odd-chain SFA [75]. Pentadecanoic acid is directly correlated with dietary intake, and the differences in this fatty acid may be attributed to differences in dietary intake between their study groups [92]. 

The observed positive associations between MUFA and MVPA in the plasma of pregnant women with GDM may be attributed to the high levels of lipogenesis and fat deposition that occur during early pregnancy. These stored lipids are later transferred to the fetus during the third trimester, and subsequently, lipolysis occurs at a higher rate. This process is crucial for the growth and development of the fetus [91]; however, the precise mechanism underlying this regulatory process has not been fully elucidated. Taken together, these studies suggest that PA is of increasing importance to maintaining optimal FA levels to ensure optimal fetal development.

AAs, too, play an indispensable role in different stages of the gestational period, including protein synthesis and various cellular functions [17]. For instance, BCAAs are crucial for embryo implantation and development through enhancing blastocyst quality through the activation of the mTOR signaling pathway. Notably, leucine is a major activator of this pathway, contributing to blastocyst activation [17]. AAs like arginine and its related counterparts, ornithine, proline, and nitric oxide, are pivotal in promoting angiogenesis and gene expression, crucial for both placental and fetal development [93]. Meanwhile, methionine and choline are essential for DNA and RNA synthesis, facilitating crucial epigenetic modifications [94]. 

Variations in amino acid concentrations might indicate an adaptive transport of AAs in response to fetal nutrition requirements or endocrine signaling [95,96]. Different gestational parent amino acid concentrations have been associated with intrauterine growth restriction [97] and gestational diabetes [98,99]. Gestational PA was associated with reduced levels of BCAAs in the third trimester of pregnancy [13]. There is previous evidence to show that BCAA metabolites, as well as the BCAAs themselves, contribute to insulin resistance and metabolic dysfunction in the non-pregnant population [100]. Therefore, the reduced levels of urinary BCAAs seen in physically active pregnant individuals compared to their non-active counterparts suggest that PA may be beneficial for the metabolic health of both the parent and fetus [12].

Another targeted metabolomics study evaluated if adopting a healthy lifestyle (e.g., diet and PA) could influence the metabolomic profile in early- and mid-pregnancy among Hispanic birthing parents who were overweight/obese. Their profiles were compared to a control group receiving normal antenatal care [74]. The intervention was developed to help these pregnant individuals meet the Institute of Medicine gestational weight gain guidelines. Plasma was collected from the participants during their glucose tolerance test (OGTT). In mid-pregnancy, “∆fast-120 min post-OGTT” was significantly lower for two long-chain monounsaturated acyl carnitines (myristoleoylcarnitine and palmitoleoylcarnitine) in the intervention group compared to the control. Smaller, non-significant decreases were also observed for amino acids, 3-HB, and bile acids, suggesting that the intervention might have had some impact on the plasma metabolites during the OGTT. These compounds are associated with FA metabolism, BCAA metabolism, and bile acid metabolism [74].

As for the cord blood metabolome, less is known. Patel et al. investigated the cord blood metabolite response to a diet and PA intervention in 343 pregnant individuals and their offspring. The authors reported no association between the intervention and cord blood metabolites, although a positive association between birth weight and weight at six months old and PCs and LPCs, primarily LPC 16:1 and LPC 18:1, was reported [73]. Numerous studies have demonstrated a correlation between PA and these metabolites in non-pregnant individuals, as shown in Table 2. These findings may be indicative of a route through which gestational parent PA impacts fetal birth weight. While data on the offspring metabolome response to gestational PA are lacking in humans, animal models have been explored. In 2022, an animal study (murine) delved into the effects of maternal exercise on various offspring tissues using targeted metabolomics [11]. Amino acids, acylcarnitines (ACs), nucleotides, and organic acids were measured and exhibited significant alterations in a tissue-specific manner as a response to parental exercise. In the liver, gestational exercise led to heightened levels of tyrosine, histidine, and phenylalanine and a decrease in pyruvate and short and long fatty acids. In skeletal muscle, there was an elevation in serine, ATP, and NADP quantities, while lactate and short ACs showed a decline. Lastly, in serum, the concentrations of valine and long ACs decreased, whereas the short AC levels increased. The upsurge in nucleotides is likely attributed to the tissue’s increased energetic demand and fatty acid oxidation in the offspring of active birthing parents. The authors postulated that the reduction in amino acids and the increase in nucleotides could contribute to enhancing insulin sensitivity and hepatic function in the offspring. Nevertheless, further research is needed to unravel the metabolic changes related to PA [11]. 

A mouse model was used to examine the effect of an eight-week exercise intervention on the development of NAFLD in the offspring of high-fat diet-fed mice [72]. Liver samples gathered from the offspring of the intervention and control groups were used for untargeted metabolomics using LC-MS. Distinct metabolites were altered in the offspring born from the exercised mice; for instance, the bile acids taurocholate and cholate sulfate, as well as Car(20:1), Car(22:0), histidine, glutamate, choline, and sarcosine, were increased. Among the reported changes, the observed increase for histidine is of great importance as histidine has been previously reported to have a protective effect against hepatic fibrosis in an experimental model [101]. Given these results, exercise in pregnancy can potentially protect the offspring against the development of liver disease caused by maternal obesity, in part by mediating changes in the offspring metabolome [72].

Considering that exercise exerts beneficial effects on lipids and amino acids in non-pregnant individuals, it is plausible that it could lead to alterations in these metabolites during pregnancy, thereby improving IR and reducing the risk of developing GDM. However, the impact of exercise on the metabolome of pregnant individuals and their offspring, particularly in low-risk pregnancies, remains largely unexplored and requires further investigation.

## 4. Future Research Focus

Our review illustrates that PA can impact the metabolomic profiles of non-pregnant as well as pregnant populations in human studies, as well as the tissue metabolomic profile of offspring in animal models. However, heterogeneity in the study design or PA exposure and small sample sizes may mask other PA-induced changes and make it challenging to map out trends among studies. Nonetheless, as discussed in the previous sections, there are both observational and interventional data in the literature demonstrating that PA can modify the levels of distinct metabolites in several biological samples. Notably, several metabolites (e.g., isoleucine, valine, lactate) and their catabolites have been linked to pregnancy and birth complications such as pre-eclampsia, gestational diabetes, and intrauterine growth restriction [102,103,104,105,106]. Accordingly, we posit that the ability of PA to regulate these metabolites and their affiliated metabolic pathways may be a potential route for how being active during pregnancy can reduce the risk of pregnancy and birth complications.

Obstetrical research has predominantly focused on investigating the negative effects of pathological exposures, such as smoking/drug use, diabetes, and obesity, on gestational parent–fetal biology. However, the impact of exposures that decrease disease susceptibility and promote health maintenance, such as PA, remains largely understudied, despite the strong association between these behaviours and a reduced risk of global diseases [106]. Investigating the metabolome response to PA during pregnancy can help in understanding the metabolic routes and mechanisms underlying the health benefits of gestational PA. Investigating the metabolome response to PA during pregnancy can help in understanding the molecular and cellular mechanisms that mediate exercise-induced therapeutic impacts. This, in turn, will contribute to the future personalization of PA interventions and the development of exercise mimetics as a novel class of therapeutics that replicate or amplify the favourable effects of PA. Identifying approaches that enhance antenatal health and mitigate risks is crucial for the well-being of multiple generations. Nevertheless, our understanding of the distinct biological adaptations, such as PA, necessary to support gestational parent and offspring development and optimize future health falls behind the awareness of other populations.

## 5. Conclusions

In summary, this literature review supports the notion that PA can have regulatory effects on the metabolome, which leads to a distinct metabolic profile that resembles the profile associated with more favourable cardiometabolic markers (e.g., better insulin sensitivity, antioxidant capacity, and endothelial functions). Although insights have been gleaned from metabolomics studies concentrating on PA and/or exercise in non-pregnant populations, pregnancy-focused studies have been limited to populations with overweight/obesity, focusing on samples from the gestational parent. Consequently, there is a need for additional research concerning the metabolomic profiles of healthy birthing parents who partake in regular PA throughout pregnancy and their corresponding placenta and offspring. The acquisition of more extensive data in this domain will contribute to a deeper understanding of how health habits during pregnancy can influence one’s lifelong well-being. By bridging this knowledge gap, we can further elucidate the metabolic mechanisms underpinning the advantages of PA in the context of pregnancy, enhance our capacity to advocate for healthy lifestyles, and identify future intervention targets. 

## Figures and Tables

**Table 1:** The main AA subgroups associated with PA.

Metabolite		Physiological Functions	Association with PA
BCAAs	Leucine	Protein synthesis [17] Energy production [17] Signaling molecules in the regulation of glucose, lipid, and protein metabolisms [17] Immune system [17] Gut health [17] Blastocyst development [17] Mammary functions [17]	Negative
	Isoleucine		
	Valine		
AAAs	Phenylalanine	Protein synthesis [18] Neurotransmitter synthesis, including dopa, dopamine, octopamine, norepinephrine, serotonin, epinephrine, etc. [18] Melatonin synthesis [18] Immune system function [19]	Negative
	Tyrosine		
	Tryptophan		
Sulfur-containing AAs	Methionine	Protein synthesis [20] Cellular assimilation of folate and folate metabolism [20] Methylation of DNA/RNA [20] Synthesis of nucleotides and thymidylate [20] Sulfur homeostasis [20] Cellular redox state regulation [20]	Negative
	Cysteine		
	Cystine		
The urea cycle AAs	Arginine	Urea cycle [21] Nitrogen balance [21] Nitric oxide synthesis [21] Hormone production stimuli such as insulin, prolactin, glucagon, and growth hormone [22]	Positive
	Ornithine		
	Citrulline		
Glucogenic AAs	Alanine	The main glucogenic AA [23] Glucose homeostasis [23] Important energy source for the central nervous system [24]	Negative
	Glutamine	Gluconeogenesis [25] Important energy source for the gut and immune cells [26] Precursor for the synthesis of glutathione [27] Nitrogen donor and nitrogen transport [25] Promotes cell growth as an anabolic signal [25]	Positive
Serine, Glycine, and Threonine		Carbohydrate utilization [28] Antioxidant pathways [28] Central nervous system’s development and functions [29]	Positive

AAs: amino acids; BCAAs: branched-chain amino acids; AAAs: aromatic amino acids; PA: physical activity.

**Table 2:** Summary of changes in amino acids in metabolomics studies on the non-pregnant population.

Study Population and Sample Size	Biosample	Study Design	Metabolomic Profiling Platform	PA Intervention or Measurement	Main Results
11 women with overweight/obesity (18–45 years of age)	Plasma	Intervention	Shotgun TOF-MS	HIIT for 6 weeks	Ornithine levels were significantly upregulated after HIIT [41].
339 healthy adults (147 women, 40–64 years of age)	Plasma	Cohort	LC-MS and GC-MS	PAEE (MET hour per day) measured by accelerometer	Distinct amino acids (e.g., valine, isoleucine, glutamate, alanine, leucine) were associated with PA, even if the intensity was low [14].
102 adults, including 47 women (30–60 years of age)	Serum	Cross-sectional	NMR	AEE derived from doubly labeled water and Sedentary Time and Activity Reporting Questionnaire	Serine, arginine, and betaine were associated with higher levels of PA [45].
5197 people, including 2120 healthy women (25–42 years of age)	Plasma	Cross-sectional	LC-MS	Self-reported questionnaires every 2–4 years	Citrulline, asparagine, glutamate, and glycine were the amino acids associated with PA [1].
12 women with PCOS (aged 25.26 ± 2.0) and 10 aged and BMI-matched healthy controls	Plasma	Intervention	LC-MS	Supervised exercise program for a 1 h session at 60% of baseline VO_2_ max, three times per week for 8 weeks	Patients with PCOS had higher concentrations of BCAAs and AAAs compared to the controls. Following an 8-week exercise intervention, the difference in amino acid concentrations no longer existed between groups for isoleucine (BCAA), phenylalanine (AAA), and tyrosine (AAA) [53].
3195 incident CVD cases and 1465 controls (30–79 years of age)	Plasma	Nested case-control	NMR	Self-reported PA and sedentary leisure time	PA had a significant inverse association with alanine and a significant positive association with glutamine. Sedentary leisure time was inversely associated with glutamine and histidine and positively associated with alanine [49].
Women with obesity, sedentary lifestyle, and insulin resistance (30–50 years of age)	Plasma	Intervention	GC–MS	Data from calorie-restricted diet + 14 wks of 4-day/wk aerobic exercise at 60–75% of maximal HR combined with data from an acute bout of exercise	Acute exercise lowered plasma amino acids as a biochemical class. Individual amino acids, including methionine, cysteine, cystine, serine, tryptophan, phenylalanine, serine, and aspartic acid, were significantly reduced during exercise [34].
1983 individuals categorized as obese and overweight (45–65 years of age)	Plasma	Cross-sectional	NMR	Self-reported questionnaire	Isoleucine and leucine levels were significantly lower in less-active individuals compared to more-active individuals. Compared to men, women had significantly lower levels of BCAAs [4].
20 adults with MASLD (13 women, 59 years of age)	Plasma, stool, and adipose tissue (AT)	Intervention	LC-MS	HIIT for 12 weeks Control: sedentary lifestyle	The samples studied had distinct metabolic profiles. AT amino acids such as arginine, leucine, phenylalanine, and proline were higher after the PA, while methyl proline was reduced in plasma. Stool samples had different leucine-contained peptides reduced in the intervention group [42].
18,897 women (aged 45 or older) free of T2D, cancer, and cardiovascular disease at baseline	Plasma	Cross-sectional	NMR	Self-reported questionnaire	Compared to the lowest quartile of LTPA, the highest quartile had significantly lower total BCAAs (1%). For individual BCAAs, the lowest vs. highest LTPA had significantly lower leucine concentrations [32].
2217 individuals (aged 41.2 ± 8.3)	Plasma	Cohort	LC/GC–MS	Self-reported questionnaire	Vigorous (but not moderate) PA was inversely associated with several metabolites independent of BMI, including glutamate [46].
sedentary adults; 14 lean and 10 obese	Plasma Stool	Intervention	LC-MS/MS	6 wks of supervised, aerobic exercise 3×/wk (60–75% heart rate reserve) for 30–60min	Serum methionine levels increased significantly by exercise irrespective of obesity status AAA and AA metabolic pathways were upregulated in obese individuals [35].

**Table 4:** Summary of metabolomics studies on the pregnant population.

Study Population and Sample Size	Sample	Study Design	Metabolomic Profiling Platform	PA Intervention/Measurement	Main Results
806 pregnant individuals (first trimester); 886 pregnant women (third trimester)	Gestational parent urine	Cohort	NMR spectroscopy	Questionnaires	PA was significantly associated with lower BCAA levels in the third trimester [12].
343 mothers with obesity and their offspring (intervention = 169, control = 174)	Cord blood serum	Randomized controlled trial and cohort	MS	An initial session with a health trainer, followed by eight weekly sessions + plus dietary advice recommending foods with a low dietary glycemic index, avoidance of sugar-sweetened beverages, and reduced saturated fats	No association between cord blood metabolites and lifestyle intervention. Phosphatidylcholines and LPCs were associated with birth weight [73].
1158 individuals with obesity (intervention = 577; control = 581)	Gestational parent serum	Randomized controlled trial	NMR spectroscopy	An initial session with a health trainer, followed by eight weekly sessions + plus dietary advice recommending foods with a low dietary glycemic index, avoidance of sugar-sweetened beverages, and reduced saturated fats	The intervention was able to decrease the magnitude of change in several metabolites, including extremely large, very large, large, and medium VLDL particles, specifically those containing triglycerides [13].
Healthy pregnant individuals in mid- and early-pregnancy (intervention = 13; control = 16)	Gestational parent plasma	Randomized controlled trial	LC-MS	A lifestyle modification intervention focused on improving PA levels, diet quality, and calorie intake in a standard care control group before 16 gestational weeks	Lifestyle intervention was able to decrease levels of acylcarnitines, amino acids, 3-hydroxybutyrate, and bile acids in mid-pregnancy [74].
600 healthy pregnant individuals (24–36 weeks)	Gestational parent serum	Cohort	Electrophoresis MS	Questionnaires	Higher PA was associated with lower 17:0 SFAs [75].
318 healthy pregnant individuals	Gestational parent plasma	Cohort	Gas chromatography system with flame ionization detection (GC-FID)	Self-administrated Pregnancy PA Questionnaire	Higher MVPA at 15–26 weeks was positively associated with total MUFAs; higher MVPA at 15–26 weeks was positively associated with oleic acid and eicosenoic acid; higher MVPA at 23–31 weeks was positively associated with palmitoleic acid [14].
Exercise-trained or sedentary male and female C57BL/6 mice	Offspring serum, liver, and triceps muscle	Animal study	LC-MS and GC-MS	Mice housed with running wheels for 2 weeks before conception and during gestation	In the liver, maternal exercise increased tyrosine, histidine, and phenylalanine and decreased pyruvate and short and long fatty acids; in the muscle, maternal exercise increased serine, ATP, and NADP and decreased short AC; in serum, maternal exercise decreased valine and long ACs, and increased short ACs [11].
High-fat, fructose, and cholesterol-fed female C57Bl/6J mice and normal diet control	Liver tissue	Animal study	LC-MS	Mice housed for voluntary exercise of at least 2 km per day for 6 weeks before conception and during gestation	Maternal exercise modified hepatic levels of taurocholate and cholate sulfate, eicosoenoylcarnitin (C20:1), behenoylcarnitine (C22:0), histidine, choline, and sarcosine [72].
